# Prion-like Spreading of Disease in TDP-43 Proteinopathies

**DOI:** 10.3390/brainsci14111132

**Published:** 2024-11-09

**Authors:** Emma Pongrácová, Emanuele Buratti, Maurizio Romano

**Affiliations:** 1 International Centre for Genetic Engineering and Biotechnology, Padriciano 99, 34149 Trieste, Italy; epp7669@nyu.edu; 2 Department of Life Sciences, University of Trieste, Via A. Valerio, 28, 34127 Trieste, Italy

**Keywords:** TDP-43, proteinopathy, prion-like, ALS, FTLD-TDP, pathological spreading, aggregation, cell-to-cell transmission

## Abstract

TDP-43 is a ubiquitous nuclear protein that plays a central role in neurodegenerative disorders collectively known as TDP-43 proteinopathies. Under physiological conditions, TDP-43 is primarily localized to the nucleus, but in its pathological form it aggregates in the cytoplasm, contributing to neuronal death. Given its association with numerous diseases, particularly ALS and FTLD, the mechanisms underlying TDP-43 aggregation and its impact on neuronal function have been extensively investigated. However, little is still known about the spreading of this pathology from cell to cell. Recent research has unveiled the possibility that TDP-43 may possess prion-like properties. Specifically, misfolded TDP-43 aggregates can act as templates inducing conformational changes in native TDP-43 molecules and propagating the misfolded state across neural networks. This review summarizes the mounting and most recent evidence from in vitro and in vivo studies supporting the prion-like hypothesis and its underlying mechanisms. The prion-like behavior of TDP-43 has significant implications for diagnostics and therapeutics. Importantly, emerging strategies such as small molecule inhibitors, immunotherapies, and gene therapies targeting TDP-43 propagation offer promising avenues for developing effective treatments. By elucidating the mechanisms of TDP-43 spreading, we therefore aim to pave the way for novel therapies for TDP-43-related neurodegenerative diseases.

## 1. Introduction

Neurodegenerative diseases characterized by the mislocalization and aggregation of TDP-43 protein aggregates are referred to as TDP-43 proteinopathies [1]. Amyotrophic Lateral Sclerosis (ALS) and Frontotemporal Lobar Degeneration with TDP-43 pathology (FTLD-TDP) are the most prominent TDP-43 proteinopathies, with TDP-43 inclusions found in approximately 97% of ALS cases [2] and 32–54% of FTLD cases [3,4]. Other conditions within this spectrum include Limbic-predominant Age-related TDP-43 Encephalopathy (LATE) [5,6], Chronic Traumatic Encephalopathy (CTE) [7], Inclusion Body Myositis (IBM) [8], Perry Syndrome [9], and Huntington’s disease (HD) [10,11].

Prions are misfolded proteins that possess the unique ability to propagate by inducing abnormal conformations in normal proteins, leading to a self-propagating cascade of aggregation [12]. While originally associated with infectious prion diseases, similar mechanisms of seeded protein aggregation have since been observed in various neurodegenerative disorders, where they contribute to disease spread [13].

Recent research has highlighted the prion-like characteristics of TDP-43, suggesting that misfolded aggregates might act as seeds, inducing conformational changes in native TDP-43 proteins and propagating pathology across neural systems [2,14,15,16]. This prion-like behavior can presumably explain the progressive nature of these diseases and their characteristic dissemination through anatomically connected brain regions [17].

Prion-like behavior is defined by the ability of misfolded protein aggregates to act as templates, inducing conformational changes in similar native proteins, which can then lead to pathology spread through interconnected neural systems [2,14,15,16]. Indeed, the properties underpinning prion-like mechanisms—such as stable, self-propagating aggregates, cell-to-cell transmission, the existence of conformational strains, selective cellular and regional vulnerability, stability and resistance to inactivation, and the toxicity of oligomers—are essential hallmarks of TDP-43 proteinopathies and provide a plausible explanation for the progressive spread of pathology observed in ALS, FTLD-TDP, and related conditions [18].

A significant body of research supports the idea that TDP-43 aggregates can act as “seeds” in this pathogenic process. Studies indicate that misfolded TDP-43 aggregates can travel between cells via exosomes or other extracellular vesicles, where they may interact with native TDP-43 proteins, promoting their misfolding and aggregation [2,14,15,16]. This cell-to-cell transmission and propagation mimic the mechanisms observed in classical prion diseases, such as Creutzfeldt–Jakob disease. However, unlike these true prions, TDP-43 proteinopathies are generally restricted to spreading within a single host. There is currently no evidence of interhost transmission of TDP-43 aggregates. Thus, TDP-43 proteinopathies lack the infectivity that characterizes prion diseases associated with cross-host transmissibility [19,20].

Experimental models—both in vitro and in vivo—have provided robust evidence of these prion-like mechanisms in TDP-43, demonstrating that aggregates introduced into healthy neuronal cultures can induce similar misfolding and aggregation in native TDP-43, leading to a progressive spread of pathology [5]. Additionally, research has identified distinct TDP-43 strains with varying structural conformations and pathogenicity, similar to the strain diversity observed in prion diseases. This strain diversity may underlie the phenotypic heterogeneity seen across TDP-43 proteinopathies, further supporting the prion-like nature of TDP-43′s intrahost spread while emphasizing the fundamental difference from transmissible prions [21].

For this reason, understanding the mechanisms underlying the prion-like spread of TDP-43 is crucial for developing targeted therapeutic strategies.

This review synthesizes current knowledge on the prion-like behavior of TDP-43, exploring its implications for disease onset, progression, and potential interventions in TDP-43 proteinopathies. The molecular basis of TDP-43 aggregation and the cellular pathways involved in its propagation will be presented, as well as the emerging evidence from in vitro and in vivo models supporting the prion-like hypothesis. Additionally, we will discuss how this conceptual framework might inform future diagnostic approaches and therapeutic strategies aimed at halting or reversing the spread of TDP-43 pathology. These strategies include targeting TDP-43 aggregation, immunotherapy, modulating autophagy and the ubiquitin–proteasome system, and addressing the exosome-mediated spread of TDP-43. In particular, addressing the mechanisms underlying the prion-like spreading of TDP-43 could lead to effective advancements in the management of these challenging conditions.

## 2. Overview of TDP-43 Structure and Functions

### 2.1. Normal Cellular Functions of TDP-43

TDP-43 is a widely conserved protein involved in many key RNA metabolism processes. Under physiological conditions, TDP-43 is predominantly localized in the nucleus, but it can shuttle between the nucleus and cytoplasm to perform its diverse functions. Its primary cellular functions include the following:

1. Transcriptional regulation: It was initially identified as a factor binding to the TAR DNA sequence of HIV-1 [22]. Subsequently, it has been shown that TDP-43 can regulate gene expression by acting as a transcriptional repressor or activator by binding the DNA directly [23,24] or by influencing HDAC6 levels [25,26].
2. RNA splicing: TDP-43 is involved in the regulation of both the constitutive and alternative splicing of pre-mRNA. It predominantly binds to UG-rich sequences in introns and promotes exon skipping or inclusion, which increases protein diversity [27] and can vary depending on cell type [28].
3. mRNA stability and transport: TDP-43 regulates mRNA stability and localization, especially transporting mRNAs to neuronal dendrites and axons [29,30,31,32,33].
4. MicroRNA processing: TDP-43 interacts with the ribonuclease complex involved in the initial step of microRNA processing (Drosha) and facilitates the production of a subset of microRNAs, thereby indirectly regulating gene expression at the post-transcriptional level [34,35,36,37].
5. Stress granule dynamics: Under cellular stress conditions, TDP-43 can localize to stress granules, which are cytoplasmic ribonucleoprotein complexes that temporarily store mRNAs and associated proteins to halt the translation of non-essential proteins [38].
6. Autoregulation: TDP-43 regulates its own expression through a negative feedback loop, binding to the 3′ UTR of its own mRNA and promoting its degradation, thus maintaining homeostatic levels of the protein [39,40,41,42].

### 2.2. Structure and Domains of TDP-43

To understand how TDP-43 performs these functions, it is important to explore its structural domains. TDP-43 is a 414-amino acid protein with a molecular weight of approximately 43 kDa. Its structure consists of several distinct domains, each contributing to its functional versatility [43,44,45,46]:

1. N-terminal domain (NTD): Comprising amino acids 1–80, the NTD is crucial for TDP-43 oligomerization and plays a role in enhancing the splicing-regulatory activity of the protein. Recent structural studies have revealed that the NTD can adopt a unique fold that promotes physiological oligomerization, which is distinct from pathological aggregation.
2. RNA Recognition Motifs (RRMs): TDP-43 contains two RRMs:
  - RRM1 (amino acids 106–175): This is the first RNA-binding domain, with a high affinity for UG-rich sequences.
  - RRM2 (amino acids 191–262): The second RNA-binding domain plays a supporting role in RNA binding and contains a nuclear export signal (NES), located between amino acids 82 and 98. The NLS is responsible for the predominantly nuclear localization of TDP-43 under normal conditions.
3. Glycine-rich C-terminal domain (CTD): This domain spans amino acids 274–414. It is intrinsically disordered and contains a prion-like domain (PrLD) [20].

A prion-like domain (PrLD) is a protein sequence motif that shares characteristics with domains found in prions, which are proteins known for their ability to misfold into self-propagating, aggregation-prone structures. These domains are often low in sequence complexity, meaning they contain repetitive patterns and are rich in certain amino acids such as glutamine (Q) and asparagine (N). In prion proteins, these domains facilitate the formation of stable aggregates that can induce similar misfolding in neighboring proteins, a property known as “prion-like” spread.

In the context of TDP-43, the prion-like domain is located within its CTD. The PrLD in TDP-43 is essential for its physiological role in forming ribonucleoprotein (RNP) granules under normal cellular conditions, which are transient, reversible aggregates involved in RNA processing and the stress response. However, under pathological conditions, this same domain enables TDP-43 to form insoluble, stable aggregates that disrupt its normal function and contribute to neurodegenerative disease progression, similar to prions.

Thus, in our interpretation, the prion-like concept in TDP-43 refers to the capacity of its PrLD to transition from functional, reversible assembly into pathological, irreversible aggregation.

The CTD is also crucial for protein–protein interactions, particularly with other heterogeneous nuclear ribonucleoproteins (hnRNPs). Interestingly, this domain is also the primary site of classic and somatic disease-associated mutations and post-translational modifications [47,48].

Structurally, the CTD can adopt various well-ordered shapes that contribute to both physiological and pathological aggregation processes. Detailed structural studies have identified distinct subdomains within the CTD: two Gly-aromatic-Ser-rich (GaroS) regions (residues 274–317 and 368–414) and an amyloidogenic core, which is divided into a hydrophobic region (residues 318–340) and a Q/N-rich region (residues 341–369) [49]. These segments play different roles in aggregation, with the GaroS regions resembling those in the FUS protein, which interact within RNA granules and contribute to hydrogel formation, suggesting a role in TDP-43 assembly dynamics [50,51].

The hydrophobic region within the CTD has been observed to adopt various structural conformations, including a helical form or Thioflavin T-positive filaments, characteristic of cross-β architecture [52,53,54]. Notably, a lipid-mimetic environment has revealed a distinct Ω-loop-helix conformation in this segment, a feature confirmed by further studies that identified a helix-turn-helix structure disrupted by disease-linked mutations [14,52]. In contrast, the adjacent Q/N-rich region exhibits a propensity for amyloid and amyloid-like aggregation and forms steric zippers that enhance TDP-43 pathogenic aggregation potential. The helix-to-sheet transition of this region supports amyloid formation and is linked to mutations and phosphorylation events that modulate these structural transitions, impacting neurotoxicity [55,56].

These structural studies suggest that the CTD adopts a spectrum of transient shapes, each capable of self-association. Mutations and post-translational modifications influence the kinetics of these transitions, potentially shifting the balance toward fibril formation or dissolution. This phenomenon, also observed in other amyloid proteins, supports the hypothesis that disease-specific “foldomes” may contribute to TDP-43 proteinopathy variability [57].

Although the CTD has been primarily associated with TDP-43 aggregation, recent findings indicate that other domains also participate in this process. The TDP-43 N-terminus, for instance, has been shown to enhance endogenous TDP-43 aggregation when fused with additional protein sequences. This aggregation causes nuclear depletion of TDP-43, disrupting its essential role in pre-mRNA splicing and leading to a functional loss without direct cellular toxicity [58]. Furthermore, the RRM2 domain also contributes to aggregation in various in vitro models [47,56,59,60]. These findings highlight the complex, multi-domain regulation of TDP-43 aggregation, underscoring the intricate interactions that govern the balance between its soluble, functional state and its pathological forms.

These findings underscore the critical role of multiple TDP-43 domains in modulating the interaction between the soluble protein and its pathological aggregates, ultimately contributing to the depletion of functional TDP-43 and the disruption of its physiological activities.

### 2.3. Post-Translational Modifications of TDP-43 and Mechanisms in Disease

TDP-43 undergoes various post-translational modifications that regulate its function, localization, and aggregation propensity [61]. The most significant modifications are listed in Table 1 and include the following:

- Phosphorylation: Hyperphosphorylation is a hallmark of pathological inclusions in TDP-43 proteinopathies. Key phosphorylation sites include serines 375, 379, 403, 404, 409, and 410. Phosphorylation can affect TDP-43 solubility, cellular localization, and interaction with other proteins [62,63,64,65,66]. This is likely an underestimation because many other sites have been recently found to be phosphorylated in post-mortem human brains from patients, and their eventual significance is very much unknown [67].
- Ubiquitination: It is observed in pathological inclusions and may play a role in protein degradation and aggregation. Both K48-linked and K63-linked polyubiquitin chains have been identified on pathological TDP-43 [68,69,70].
- Acetylation: Lysine acetylation at specific sites (K84 and K136) in TDP-43 affects its nuclear localization, RNA binding, and phase separation. Acetylation at K136 can disrupt RNA binding and promote aggregation, while deacetylation by SIRT1 can reduce aggregation [71,72].
- SUMOylation: SUMOylation (post-translational modification involving the attachment of SUMO—Small Ubiquitin-like Modifier—proteins) of TDP-43 occurs at lysine 136 (K136) located within the RRM1 domain. This post-translational modification has significant effects on TDP-43 functions, including its ability to regulate RNA splicing, its distribution within cellular compartments, and its association with stress granules [73].
- O-GlcN-Acylation: O-GlcNAc transferase (OGT) directly modifies TDP-43 by adding O-GlcNAc (N-acetylglucosamine) to specific amino acid residues. Primarily, OGT targets threonine residues at positions 199 (T199) and 233 (T233) within the TDP-43 protein. This event is likely crucial for regulating TDP-43 function and stability, especially in the context of neurodegenerative diseases [74].
- Cysteine oxidation: TDP-43 oxidation at specific cysteine residues (C173 and C175), located in the RRM1, can lead to protein aggregation and disease progression [75].
- Proteolytic cleavage: In pathological conditions, TDP-43 can be cleaved by caspases (3, 7, and 4) and asparaginyl endopeptidase (AEP). Caspases 3 and 7 generate 35 kDa and 25 kDa C-terminal fragments during apoptosis [76]. Caspase-4 cleavage after N174 initiates a cascade leading to further fragmentation and clearance [77]. AEP cleaves TDP-43 at multiple sites, including N291 and N306, generating ~35 kDa and 32 kDa fragments [78]. A novel 15 kDa fragment, with varying prominence across species, has also been identified [79]. These cleavage events can contribute to the accumulation of insoluble, phosphorylated TDP-43 species, a hallmark of certain neurodegenerative disorders.
- Argynilation. TDP-43 has been recently described to bind tRNA^Arg^ in vitro [80] and the Ate1 co-factor LIAT. Arginyltransferase Ate1 facilitates the degradation of TDP-43 protein fragments by transferring an arginyl group from tRNA^Arg^ to the C-terminal region of these fragments. This modification targets the fragments for degradation by the ubiquitin–proteasome system, thereby maintaining TDP-43 protein quality control.
- Citrullination and monomethylation of R293. These modifications have not yet been functionally characterized but have been identified in TDP-43 fibrils from the brains of three individuals with the most common type of FTLD-TDP, type A [81].

These structural features and post-translational modifications contribute to the TDP-43 propensity for aggregation and potential prion-like behavior in pathological conditions, forming the basis for investigating the mechanisms of TDP-43 misfolding, aggregation, and spread in neurodegenerative diseases.

## 3. TDP-43 Proteinopathies

TDP-43 proteinopathies are neurodegenerative disorders marked by TDP-43 inclusions in neurons and glial cells. Despite their common molecular pathology, these diseases exhibit diverse clinical manifestations. The most prominent TDP-43 proteinopathies include ALS, FTLD-TDP, HD, and other associated conditions, such as LATE, CTE, AD, IBM, and Perry Syndrome.

### 3.1. Amyotrophic Lateral Sclerosis

ALS is the most studied TDP-43 proteinopathy. This neurodegenerative disorder primarily affects motor neurons in the brain, brainstem, and spinal cord. ALS has a global prevalence of approximately 2–3 per 100,000 individuals. Clinically, it presents with progressive muscle weakness, atrophy, fasciculations, and ultimately paralysis, with cognitive impairment occurring in a subset of patients. TDP-43 pathology is observed in about 97% of ALS cases, including both sporadic and familial forms. Genetic mutations in the gene encoding the TDP-43 protein (*TARDBP*) contribute to roughly 5% of familial ALS cases, while other genes such as *C9ORF72*, *SOD1*, and *FUS* are also implicated in the pathogenesis. The median survival following symptom onset is 3–5 years, though some patients experience longer survival [82].

### 3.2. Frontotemporal Lobar Degeneration

FTLD-TDP causes early onset dementia, with degeneration of the frontal and temporal lobes. It accounts for roughly 45% of all FTLD cases. Clinically, FTLD-TDP presents with either a behavioral variant, marked by personality changes and social dysfunction, or language variants, where speech and language impairments dominate. TDP-43 pathology in FTLD-TDP is categorized into four distinct patterns (Types A–D), each associated with different clinical and genetic subtypes [3,83,84,85]. Genetic mutations in the gene encoding progranulin (*GRN*), *C9ORF72*, and the gene encoding the valosin-containing protein (*VCP*) are linked to FTLD-TDP [86,87]. There is also notable overlap between ALS and FTLD, with some patients exhibiting symptoms of both diseases, emphasizing their clinical and pathological intersection [88,89].

### 3.3. Huntington’s Disease

Beyond ALS and FTLD-TDP, TDP-43 is implicated in several other neurodegenerative disorders, including Huntington’s disease. 

Although the disease is primarily driven by the mutant huntingtin protein, increased levels and abnormal accumulation of TDP-43 in the cytoplasm have been reported in HD. These pathological changes correlate with the severity of cognitive, motor, and behavioral symptoms in HD patients [10,11,90,91]. The abnormal accumulation of TDP-43 in the cytoplasm, where it forms aggregates with the mutant huntingtin protein, is thought to contribute significantly to neuronal dysfunction and degeneration in HD [10,11].

Moreover, the most common type of mRNA modification, m6A methylation, is reduced in mRNAs that are abnormally expressed in the striatum of HD mouse models, particularly at sites close to TDP-43 binding sites. This evidence suggests that a combination of decreased TDP-43 function and altered m6A modification may contribute to the abnormal splicing and expression of genes in HD [92,93].

### 3.4. Other TDP-43-Associated Disorders

Other neurodegenerative disorders have been linked to TDP-43 pathology. Limbic-predominant Age-related TDP-43 Encephalopathy (LATE) is a condition resembling Alzheimer’s disease, primarily affecting the limbic structures in older adults and contributing to cognitive decline [5,6]. Chronic Traumatic Encephalopathy (CTE), associated with repeated head trauma, presents with cognitive decline, behavioral changes, and motor symptoms. TDP-43 pathology in CTE often co-occurs with tau pathology, though its precise role remains under investigation [7]. In Alzheimer’s disease, TDP-43 inclusions are found in 30–70% of cases, particularly in the limbic regions, and are linked to more rapid cognitive decline, suggesting that TDP-43 may represent a distinct subtype or comorbid pathology in AD [6,94].

TDP-43 pathology is not limited to the more well-known neurodegenerative disorders. It also plays a role in less frequent but equally important conditions. For instance, Inclusion Body Myositis (IBM), an uncommon inflammatory muscle disease, exhibits TDP-43 abnormalities in the affected muscle tissue [8,95,96]. Similarly, Perry Syndrome is a rare disorder caused by DCTN1 mutations and characterized by parkinsonism and additional symptoms such as depression and weight loss, with TDP-43 pathology observed in specific brain regions such as the brainstem nuclei and substantia nigra [97,98,99]. Another pathology where TDP-43 has been shown to be mislocalized is in the Purkinjie neurons of Niemann–Pick Type C patients [100]. These examples highlight the diverse range of conditions impacted by TDP-43 dysfunction.

TDP-43 pathology across diseases suggests common mechanisms, thus opening opportunities for unified therapeutic strategies. Investigating the prion-like behavior of TDP-43 could provide valuable insights into how these diseases spread and evolve.

## 4. Pathological Aggregation and Mislocalization of TDP-43

TDP-43 aggregation and mislocalization are key factors in neurodegenerative diseases, driven by its prion-like behavior. Understanding these processes is crucial for developing strategies to mitigate the toxic effects of TDP-43 and slow disease progression.

### 4.1. TDP-43 Aggregation Mechanisms

**Figure 1:**
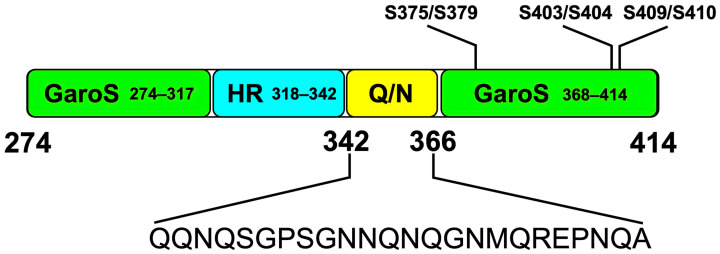
The low-complexity domain of TDP-43. The structural elements of the glycine-rich C-terminal domain (CTD) within the low-complexity domain (LCD) of TDP-43 (amino acids 274–414) are depicted. Detailed structural analyses have identified distinct subdomains within the CTD: two Gly-aromatic-Ser-rich (GaroS) regions (residues 274–317 and 368–414), an amyloidogenic core divided into a hydrophobic region (HR, residues 318–342), and a Q/N-rich region (residues 341–369). The potential phosphorylation sites (S375/379, S403/S404, and S409/S410) that may influence aggregation are also indicated.

Recent studies have highlighted important insights into the aggregation dynamics and structural properties of TDP-43, emphasizing the complexity of its pathology [101,102]. The C-terminal low-complexity domain (LCD, aa 274–414) enables TDP-43 to form oligomeric structures that can shift from a liquid to a more solid-like state, particularly in response to cellular stress (Figure 1). 

These oligomers, which form spheroidal and ring-like shapes, are toxic to neurons [103]. Once aggregation starts, it recruits more TDP-43 molecules, creating a self-perpetuating cycle like prion diseases. The intrinsically disordered prion-like domain (PrLD) of TDP-43 is critical for its assembly dynamics. It is important to clarify that the term “prion-like domain” is not universally accepted or defined. However, in the context of TDP-43, it refers to the ability of specific regions of the protein, particularly the LCD, to facilitate the formation of prion-like seeds. 

The prion-like domain (PrLD) of TDP-43, which is intrinsically disordered, plays a pivotal role in its assembly and aggregation dynamics. Aliphatic and aromatic residues within this domain are essential in driving the transition of TDP-43 from liquid-like condensates to more solid amyloid structures, both of which are integral to its normal and pathological roles [104]. A key aspect of these aggregates is phosphorylated TDP-43 (pTDP-43), which has been observed to accelerate aggregation in healthy cells and promote neurodegeneration [72]. Intriguingly, recent studies have also suggested a potentially protective role of hyperphosphorylation in preventing aggregation, creating a paradox regarding the exact function of pTDP-43 [105]. 

A central hypothesis to explain the paradoxical effects of pTDP-43 is based on its role in phase separation and aggregation dynamics. TDP-43 is known to undergo liquid–liquid phase separation (LLPS), which allows it to form transient, liquid-like condensates in the cellular environment. These condensates are generally dynamic and reversible, facilitating normal cellular functions. Phosphorylation of TDP-43 could modulate these phase transitions, influencing whether TDP-43 remains in a soluble, liquid-like state or progresses to a more solid, aggregated form. For instance, hyperphosphorylation at multiple sites, particularly in the C-terminal region, may disrupt the internal protein–protein interactions within these condensates, thereby enhancing their solubility and liquidity. This disruption could prevent the pathological transition from liquid-like droplets to solid, amyloid-like aggregates, which are known to be toxic to cells. Thus, in certain contexts, hyperphosphorylation may act as a cellular mechanism to mitigate aggregation, safeguarding TDP-43 from adopting pathogenic conformations.

On the other hand, phosphorylation at specific sites or under particular conditions may stabilize TDP-43 in conformations more prone to aggregation, thus promoting pathological effects. When phosphorylation is limited or occurs at sites that favor aggregation, TDP-43 may escape the solubilizing effects of hyperphosphorylation and instead form solid, insoluble inclusions. These inclusions, unlike liquid-like condensates, are challenging for cellular systems to manage and can accumulate to toxic levels, contributing to neurodegeneration. Therefore, the effect of pTDP-43 may depend on the specific phosphorylation pattern, with hyperphosphorylation acting as a modulator that influences whether TDP-43 transitions into harmful aggregates.

Then, other factors may also contribute to the complex behavior of pTDP-43. In response to cellular stress, TDP-43 frequently localizes to membrane-less organelles, like stress granules. Phosphorylation could affect the incorporation of TDP-43 into these structures. For example, extensive phosphorylation might prevent TDP-43 from stably associating with stress granules, thereby reducing its propensity for aggregation in these compartments. This process may represent a protective adaptation under stress, especially in neurons, where reducing TDP-43 aggregation could play a vital role in cell survival.

Finally, phosphorylation introduces negative charges to TDP-43, which could enhance its solubility by promoting favorable interactions with water molecules and reducing its tendency to form protein–protein interactions. Molecular simulations support this idea, showing that phosphomimetic modifications of TDP-43 can increase its solubility, likely explaining how phosphorylation might sometimes counteract aggregation.

In conclusion, as previously mentioned, it should be considered that, while the LCD has been shown to be crucial for aggregation, other regions of the protein, such as the N-terminal domain, also play important roles in TDP-43 self-assembly and aggregation. The NTD of TDP-43 is essential for trapping protein clumps and, as a result, the protein loses its functionality. This was observed when the generation of a cellular model expressing multiple copies of the Q/N-rich domain of TDP-43 showed that these tandem repetitions are able to induce aggregation of endogenous TDP-43 only when this sequence was present [106].

### 4.2. Cytoplasmic Mislocalization and RNA Metabolism

Cytoplasmic mislocalization of TDP-43 is a hallmark of neurodegenerative conditions, such as ALS and FTLD. Under pathological conditions, TDP-43 forms insoluble inclusions in the cytoplasm that disrupt its nuclear functions, leading to impaired RNA metabolism and cellular dysfunction [107,108]. These aggregates are ubiquitinated, indicating protein quality control failure and linked to nucleocytoplasmic transport defects [109,110], and in general, TDP-43 mutations have been shown to worsen cytoplasmic aggregation and toxicity [111,112,113,114].

### 4.3. Liquid–Liquid Phase Separation

In the process of aggregation, liquid–liquid phase separation (LLPS) plays a crucial role in TDP-43 pathology [115]. While TDP-43 normally undergoes reversible LLPS to form dynamic nuclear structures that help cells respond to stress [116], pathological conditions can lead to dysregulated LLPS and harmful cytoplasmic aggregates [117]. The transition from a liquid-like to a solid state is influenced by various post-translational modifications, such as methionine oxidation, which alters the structural properties of the TDP-43 prion-like domain [103] or nuclear RNA levels that help to reduce excessive LLPS [118].

### 4.4. Detecting Prion-like Seeding and Cross-Seeding

Recent advances in understanding TDP-43 prion-like behavior have led to the development of the TDP-43 seeding amplification assay (TDP43-SAA) for detecting TDP-43 aggregates in clinical samples [119]. This assay has also shown promise in identifying TDP-43 pathology in olfactory mucosa from FTLD-TDP patients, demonstrating high accuracy in differentiating cases with TDP-43 neuropathology from controls [120].

This technique could serve as a valuable tool for identifying and monitoring TDP-43 pathology in living patients. Interestingly, recent studies have shown that the misfolded prion protein (PrP) can cross-seed TDP-43, leading to its aggregation and subsequent loss of physiological function. Interestingly, this interaction has been demonstrated in both cellular models and human brain samples, elucidating a novel pathway by which prion aggregates could disrupt TDP-43 functionality [121].

## 5. Evidence for Prion-like Spreading of TDP-43

The prion-like behavior of TDP-43 is supported by multiple lines of evidence, ranging from seed-dependent aggregation to variant-like behavior and regional progression in the nervous system (Table 2).

Taken together, these characteristics closely resemble those of traditional prion diseases, strengthening the hypothesis of TDP-43’s prion-like nature in neurodegenerative disorders.

### 5.1. Seed-Dependent Aggregation

In vitro experiments have demonstrated that pre-formed, insoluble TDP-43 aggregates from diseased brains can induce aggregation of endogenous TDP-43 in cultured cells. For instance, introducing fibrillar TDP-43 aggregates into HEK293T cells led to the formation of similar aggregates, resembling those seen in ALS patients [122]. Similarly, exposing human neuroblastoma cells to different TDP-43 variants resulted in insoluble inclusions that retained the properties of the original aggregates [15]. This evidence supports TDP-43’s ability to self-replicate its pathological features, a key characteristic of prion-like behavior.

### 5.2. Cell-to-Cell Transmission

**Figure 2:**
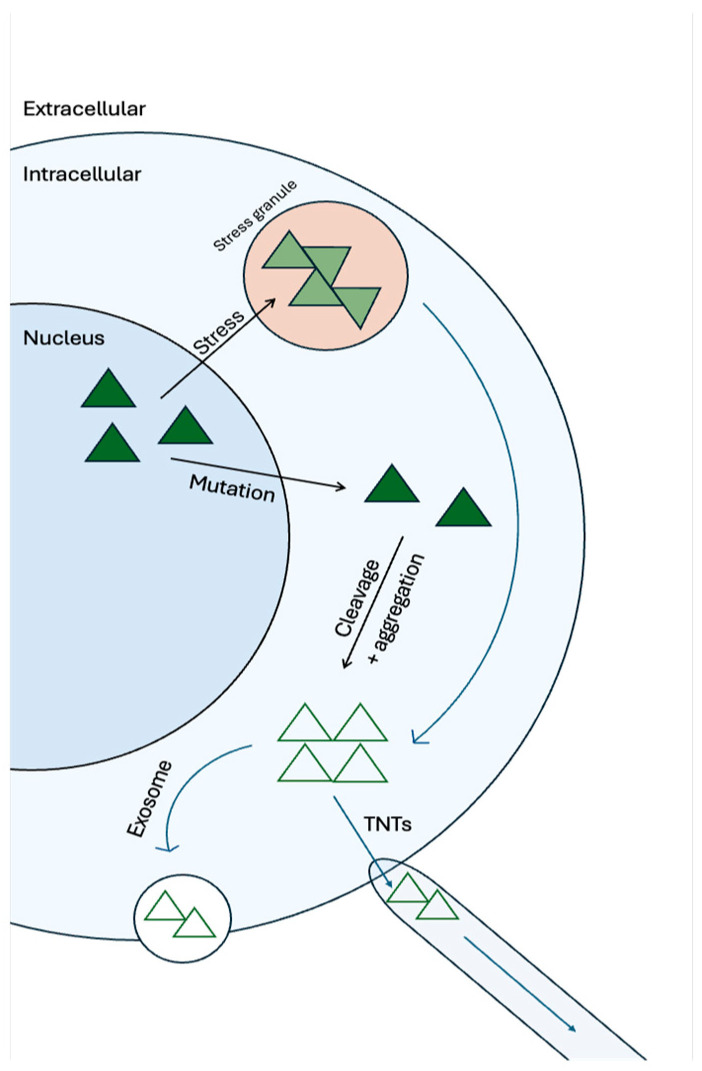
Intracellular and extracellular processes leading to the prion-like spread of TDP-43. The interplay between intracellular and extracellular factors that contribute to the prion-like spread of TDP-43 pathology is highlighted. Within cells, TDP-43 can form aggregates in response to cellular stress, undergo fragmentation and structural changes, and become mislocalized to the cytoplasm. Mutations and cleavage events can further exacerbate aggregation. These intracellular processes set the stage for the propagation of TDP-43 pathology. Extracellularly, TDP-43 aggregates can be released into the surrounding environment through exosomes, small vesicles that facilitate intercellular communication. These exosomes can transport TDP-43 aggregates to neighboring cells, potentially spreading the pathology to new regions of the brain or other tissues.

TDP-43 aggregates have been shown to spread between cells, further supporting their prion-like nature. Serial passage experiments have demonstrated that aggregates can be effectively transferred from one culture to another, increasing aggregation in recipient cells. Notably, oligomeric forms of TDP-43 can disseminate through microvesicles or exosomes, facilitating the intercellular spread of TDP-43 pathology [123]. Figure 2 shows how both intracellular and extracellular processes could lead to prion-like spread of TDP-43.

In addition to these primarily biochemical and cellular experiments, time-course microscopy has captured the intercellular propagation of phosphorylated and ubiquitinated TDP-43 aggregates in stressed cells, illustrating how TDP-43 can spread beyond its initial cellular environment [124].

### 5.3. Regional Progression in the Nervous System

TDP-43 prion-like behavior is further supported by its regional progression in the nervous system, similar to traditional prion diseases. Indeed, it has been well known that TDP-43 aggregates can propagate along interconnected neural networks, resulting in a characteristic spread of pathology across various brain regions [125]. The observation that TDP-43 pathology in ALS can spread progressively along the nervous system supports the idea that TDP-43 behaves like a prion protein [126].

### 5.4. Strain-like Behavior

TDP-43 aggregates show strain-like behavior, a characteristic commonly associated with prions, where different aggregate conformations influence their ability to propagate and their pathogenic potential [127]. Various strains of TDP-43, derived from the brains of patients with ALS and FTLD, have been shown to possess unique biochemical properties and structural features that significantly affect their aggregation and transmission patterns. Studies have revealed that distinct FTLD subtypes exhibit TDP-43 assemblies with varying neurotoxicity and seeding capacities, correlating with differences in disease progression rates [128]. The C-terminal domain of TDP-43 is particularly important for aggregation, often adopting disordered conformations that facilitate the formation of β-sheet-rich oligomers. Mutations within this region can alter its structure, influencing its propensity to aggregate [53].

Moreover, these different TDP-43 strains from FTLD subtypes have been found to induce diverse aggregate morphologies and spreading behaviors both in vitro and in vivo, further supporting the concept that strain-specific properties underlie the heterogeneity in FTLD-TDP pathology [129]. Finally, it was observed that when pathological TDP-43 aggregates were used as seeds, they produced various forms of cellular inclusion bodies, closely mirroring the strain-dependent propagation seen in prion variants [15,126,130]. Taken together, this variability could underscore the role of TDP-43 in driving the clinical heterogeneity observed in neurodegenerative disorders associated with its pathology.

### 5.5. Tunneling Nanotubes and Extracellular Vesicles

Further strengthening the prion-like spreading hypothesis, a recent study utilizing a cerebrospinal fluid (CSF)-cultured cell model provided direct evidence for the transmission of TDP-43 aggregates.

In a study examining glioma cells exposed to CSF from patients with ALS-FTD, researchers observed that TDP-43 protein was mislocalized and formed aggregates within the cells. Exosomes derived from ALS-FTD CSF were found to contain elevated levels of TDP-43 C-terminal fragments (CTFs), suggesting a potential mechanism for the spread of this pathological protein. Furthermore, exposure to ALS-FTD CSF led to the formation of tunneling nanotubes (TNTs) and exosomes, which facilitated the transfer of TDP-43 aggregates between cells [131].

A separate study on sporadic ALS patients revealed that lymphoblastoid cells secreted pathogenic forms of TDP-43 into the extracellular space, where they were transported by extracellular vesicles. These vesicles could spread TDP-43 pathology to healthy cells, including lymphoblasts, myoblasts, and neuroblastoma cells.

TNTs were also observed in pathological cells and were involved in the transfer of TDP-43. A novel Casein Kinase 1 (CK-1) inhibitor, IGS2.7, was shown to prevent the spread of TDP-43 pathology by targeting its phosphorylation and restoring TDP-43 homeostasis in patient-derived cells [132].

### 5.6. Interaction with Cellular Prion Protein

The cellular prion protein (PrPC) has been identified as a receptor that increases the uptake of TDP-43 fibrils, enhancing their neurotoxic effects. Experiments indicate that cells overexpressing PrPC exhibit heightened TDP-43 fibril internalization, which correlates with increased cellular toxicity [133]. This finding not only provides further evidence for the prion-like nature of TDP-43 but also highlights a potential mechanism for its spread and toxicity in neurodegenerative diseases.

## 6. Mechanisms of TDP-43 Prion-like Propagation

The propagation of TDP-43 aggregates within the nervous system involves complex mechanisms of cellular uptake, release, and intercellular transmission summarized in Table 3. Understanding these processes is crucial for developing targeted therapies to limit TDP-43 spread in neurodegenerative disorders.

### 6.1. Cellular Uptake and Release

The first mechanism is represented by the possibility that pathological TDP-43 could enter neighboring cells primarily through membrane fusion or endocytosis, transferring aggregates that trigger the misfolding and aggregation of native TDP-43 in recipient cells (Figure 2). Recent research has shown that TDP-43 intercellular transmission is facilitated when its N-terminus is preserved and occurs without the involvement of extracellular vesicles, necessitating close cellular proximity for effective spread [134,135].

### 6.2. Intercellular Transport Mechanisms

Different mechanisms seem to contribute to TDP-43 dissemination:

- Axonal transport: TDP-43 aggregates can be transported along axons, facilitating their spread between interconnected neurons [136,137].
- Extracellular vesicle secretion: TDP-43 can be released and taken up by cells through exosomes and microvesicles [138].
- Macropinocytosis: This process allows for the uptake of large particles, including protein aggregates [139].

These mechanisms enable neuron-to-neuron and neuron-to-glia communication, promoting the spread of TDP-43 pathology [124,140].

### 6.3. Role of Glial Cells

Both astrocytes and microglia, which express TDP-43, are thought to play a significant role in the prion-like propagation of TDP-43 pathology. These glial cells can uptake TDP-43 aggregates from neurons and subsequently release them back into the extracellular environment, facilitating a broader distribution of pathology [5,137,141]. This process of transmission from glial cells to neurons contributes to non-cell autonomous toxicity, wherein glial activation exacerbates neuronal degeneration associated with TDP-43 accumulation.

Recent studies further underscore the complexity of TDP-43 spread by demonstrating that TDP-43 strains derived from distinct FTLD-TDP subtypes exhibit unique seeding and spreading properties in glial cells [129]. Specifically, brain-derived TDP-43 extracts from various FTLD-TDP subtypes have been shown to induce distinct aggregate morphologies and propagation patterns, both in vitro and in vivo. This suggests that structural heterogeneity among TDP-43 strains plays a crucial role in modulating the prion-like behavior of TDP-43 within glial cells.

These findings parallel the strain diversity observed in PrP pathologies, where prion strain-specific conformations lead to distinct aggregation and propagation characteristics. Similarly, TDP-43 strain-specific structures appear to drive differing aggregation behaviors and patterns of pathology spread, which may contribute to the phenotypic heterogeneity seen across TDP-43 proteinopathies.

### 6.4. Disruption of Axonal Transport

Interestingly, like the prion protein (PrPc) in prion diseases, TDP-43 aggregates may disrupt axonal transport via casein kinase 2 (CK2) activation. This disruption may contribute to the dying-back neurodegeneration observed in TDP-43 proteinopathies, emphasizing the importance of axonal transport in neuronal health. CK2 might represent a potential therapeutic target to mitigate the effects of TDP-43 on neuronal transport, as suggested by studies linking CK2 activation to pathogenic processes in various prion diseases [142].

### 6.5. Sequential Progression of Pathology

Although the evidence for transferring TDP-43 through conditioned medium is not as definitive as it is for SOD1 [143], TDP-43 aggregates have been observed to spread in a sequential manner similar to prions, aligning with the sequential progression of disease symptoms [144]. This pattern of spread further supports the prion-like nature of TDP-43 propagation in neurodegenerative disorders.

## 7. Experimental Models Supporting Prion-like Spreading

Research on TDP-43 prion-like spread has advanced through experimental models, especially in vitro and in vivo systems that allow for detailed analysis and have played an essential role in elucidating the mechanisms underlying TDP-43 aggregation and spreading. From a methodological point of view, researchers have employed cell culture systems to rigorously examine the seeding and aggregation properties of TDP-43. 

For example, it has been demonstrated that the introduction of pre-formed, insoluble TDP-43 aggregates into HEK293T cells can activate the aggregation of endogenous TDP-43 [122]. This process resulted in the formation of detergent-insoluble inclusions reminiscent of the pathological features observed in affected patients. In 2013, distinct TDP-43 variants from ALS and FTLD patients were identified, strengthening this evidence [15]. When introduced to cultured cells, these aggregates induced specific aggregation patterns that mirrored the original seeds, demonstrating TDP-43’s ability to propagate in a seed-dependent manner.

Building on these in vitro findings, organoid [145] and animal models [137,146] have further supported the prion-like spread of TDP-43. Transgenic mice engineered to express human TDP-43 have been crucial for studying the consequences of injecting pathological brain extracts. For example, it was demonstrated that the administration of FTLD patient-derived extracts into the brains of these transgenic mice resulted in the emergence of new TDP-43 inclusions [137]. Over time, these inclusions exhibited a propagation pattern across connected neuroanatomical regions, providing a clear illustration of the time-dependent spread of TDP-43 pathology consistent with cell-to-cell transmission dynamics. Since then, other studies have shown that injecting pre-formed TDP-43 fibrils into specific brain regions triggers widespread accumulation of phosphorylated TDP-43 (pTDP-43) in both the injection site and contralateral areas [146]. This observation supports the hypothesis that axonal transport may serve as a critical mechanism through which TDP-43 pathology is disseminated throughout the nervous system.

In addition to experimental models, human pathological studies confirm the prion-like spread of TDP-43 in clinical settings. In particular, detailed observations from post-mortem analyses of brains from individuals diagnosed with ALS and FTLD-TDP reveal a characteristic and stereotyped progression of TDP-43 pathology across interconnected brain regions. The similar aggregate forms identified in various brain areas imply that these aggregates may function as seeds for further aggregation, thereby facilitating the spread of pathology through neural networks. 

Additionally, there exists a well-documented correlation between the pattern of TDP-43 accumulation and clinical characteristics, such as symptom onset and disease progression [147,148,149,150]. Recent studies suggest that, similar to prion proteins, TDP-43 aggregates exhibit structural heterogeneity that may significantly influence their seeding, spreading, and toxicity. Distinct TDP-43 strains—derived from various neurodegenerative disease contexts like ALS and FTLD—demonstrate unique conformational properties and aggregation patterns, leading to variations in disease pathology and progression. This structural diversity among TDP-43 aggregates resembles the prion strain differences observed in PrP-related diseases, where distinct conformations correlate with specific clinical outcomes and patterns of neurodegeneration.

The relationship between TDP-43 structural variation and clinical characteristics supports the concept that TDP-43 behaves analogously to prion proteins, with strain-specific features that can propagate pathology and influence disease phenotype. Understanding this structural heterogeneity is crucial, as it highlights the potential for different aggregate forms of TDP-43 to drive distinct mechanisms of neurodegeneration across TDP-43 proteinopathies.

A deeper understanding of these intriguing mechanisms is vital for the development of targeted therapeutic strategies aimed at mitigating the spread and impact of TDP-43 pathology in affected individuals, offering hope for interventions that could alter the disease course or alleviate its effects.

## 8. Implications for Disease Progression and Diagnosis

The prion-like spread of TDP-43 proteinopathy across different neurodegenerative diseases offers crucial insights into disease progression and clinical outcomes. Misfolded TDP-43 spreads in a manner that mirrors prion-like mechanisms, where the pathology propagates across brain regions, worsening over time. This progressive spread underscores the importance of staging TDP-43 pathology to better understand its role in diseases, such as ALS, FTD-TDP, and LATE-NC [151]. Distinct staging systems have been proposed, mapping the accumulation and regional distribution of TDP-43 to specific clinical syndromes. These systems reveal how TDP-43 pathology advances throughout the brain and correlate with symptom severity, allowing for a deeper understanding of disease progression and complexity [151,152,153].

To enhance the accuracy of these staging models, data-driven approaches are increasingly employed, incorporating quantitative assessments to classify different TDP-43 proteinopathies. Such approaches have shown remarkable success in differentiating between neurodegenerative conditions based on the severity and regional spread of TDP-43 pathology. This will improve diagnostic precision and facilitate patient stratification and the development of targeted clinical interventions.

In addition to the development of staging systems, the identification of biomarkers to track TDP-43 pathology plays a pivotal role in both early diagnosis and monitoring disease progression. Traditionally, the diagnosis of TDP-43-related disorders such as ALS and FTLD-TDP has relied heavily on post-mortem histopathological examination, which limits early detection and therapeutic intervention [154,155,156]. This underscores the urgent need for minimally invasive biomarkers that can detect TDP-43 alterations in living patients, enabling earlier diagnosis and better disease management.

Recent research in biomarker discovery has focused on biofluids such as cerebrospinal fluid (CSF) and blood [157], which can be accessed relatively easily and non-invasively, or even cell fragments, such as platelets [158] and extracellular vesicles [159]. In particular, protein-based biomarkers, such as specific TDP-43 fragments or phosphorylated isoforms, hold promise for clinical use [157]. The detection of TDP-43 changes, including its aggregation, mislocalization, or post-translational modifications, in these biofluids could provide valuable insights into the presence and progression of TDP-43 pathology [160,161]. These biomarkers could then be used to monitor disease progression in real time, offering a dynamic view of the disease’s evolution and helping to assess the efficacy of therapeutic interventions. Moreover, longitudinal biomarker data could help track individual patient trajectories, potentially leading to more personalized treatment approaches. As research in this field advances, we are moving closer to the possibility of using biofluid-based biomarkers for the routine clinical monitoring of TDP-43 proteinopathies, improving diagnostic accuracy and facilitating the development of targeted therapies.

Moreover, neuroimaging techniques, such as positron emission tomography (PET), have become vital in visualizing the in vivo spread of TDP-43 aggregates. These technologies offer insights into the spatial distribution and progression of TDP-43 pathology [162]. Notably, the PET tracer [18F]AV-1451, initially developed for tau imaging, has also shown an ability to bind to brain regions affected by TDP-43 in semantic dementia [163]. This opens new possibilities for tracking TDP-43-related neurodegeneration in real time, linking the prion-like spread of TDP-43 to clinical symptoms.

The development of specialized PET tracers to detect TDP-43 pathology in vivo represents a significant leap forward, particularly for monitoring the effects of emerging therapies aimed at slowing or halting disease progression. As these imaging techniques evolve, they are expected to enhance diagnostic accuracy and provide valuable insights into the trajectory of the disease, potentially transforming the management and treatment of TDP-43-related neurodegenerative conditions.

## 9. Therapeutic Implications

Targeting the prion-like spread of TDP-43 aggregates represents a critical avenue for developing future therapies against TDP-43 proteinopathies (Table 4).

### 9.1. Targeting TDP-43 Aggregation

Inhibiting TDP-43 aggregation and spread is key to reducing its toxic effects and altering disease progression. One promising therapeutic strategy involves small molecule inhibitors designed to disrupt TDP-43 aggregation. For example, acridine-imidazole derivatives like AIM4 have shown effectiveness in reducing TDP-43 aggregates in both in vitro and cellular models by interfering with the molecular pathways that facilitate its misfolding [164,165]. Alternatively, structural modifications like C-terminal substitutions stabilize TDP-43, preventing aggregation while preserving its normal function [166]. These strategies are vital as they not only address the aggregation process but also seek to preserve TDP-43’s normal activity, potentially halting or reversing disease progression.

### 9.2. Immunotherapy

Immunotherapy offers another innovative approach, directly targeting the prion-like spreading of TDP-43 aggregates. Antibodies or single-chain variable fragments are engineered to recognize and bind specifically to the pathological forms of TDP-43 [167,168]. These antibodies can be delivered into the central nervous system using viral vectors, where they can disrupt harmful protein interactions and reduce neuroinflammation, thereby interfering with the spread of aggregates while sparing its physiological forms to maintain normal cellular functions.

While many gene therapy approaches for TDP-43 proteinopathies focus on reducing overall TDP-43 levels or correcting its mislocalization, some strategies are being developed to prevent the propagation of misfolded TDP-43 from cell to cell.

One promising approach involves the use of engineered intrabodies, which are intracellularly expressed antibody fragments. In vitro studies have provided compelling evidence for the efficacy of intrabody therapy in reducing TDP-43 aggregates. The 3B12A intrabody, derived from a monoclonal antibody targeting aggregated TDP-43, has shown remarkable success in binding and eliminating misfolded TDP-43 from cultured cells. This is important given the harmful effects of TDP-43 aggregates on neurons [169]. Subsequently, the administration of 3B12A intrabodies in transgenic mice expressing mutant forms of TDP-43 has resulted in a significant reduction in aggregate formation and associated neurotoxicity [170]. These findings suggest that intrabody therapy could offer a promising avenue for improving motor function and overall survival in patients affected by TDP-43-related disorders.

### 9.3. Targeting the Autophagy and UPS Components

Recent research has highlighted the potential of genetic manipulation strategies targeting the ubiquitin–proteasome system (UPS) and autophagy pathways as a promising therapeutic approach for TDP-43 proteinopathies. These strategies aim to enhance the degradation of pathological TDP-43, potentially reducing the spread of TDP-43 aggregates. A key focus of these approaches is the manipulation of essential UPS components that may be dysfunctional or depleted in ALS and FTD. By enhancing the efficiency of proteasomal TDP-43 degradation, these strategies could reduce the pool of misfolded TDP-43 available for prion-like propagation.

For example, one promising avenue involved the overexpression of wild-type UBQLN2, a protein involved in delivering ubiquitinated proteins to the proteasome. Studies in human neuroglioma cells have shown that co-transfection with wild-type UBQLN2 decreases the abundance of both full-length and C-terminal fragment (CTF) TDP-43 species [171]. Importantly, this effect was achieved without detectable changes in global proteasomal activity, suggesting a specific enhancement of TDP-43 degradation.

A similar approach targeted the ubiquitin pool itself. In cellular models demonstrating UPS impairment due to ubiquitin sequestration, overexpression of ubiquitin has been shown to restore the free ubiquitin pool [172]. This restoration enhanced UPS-mediated degradation of cytoplasmic TDP-43 inclusions, potentially reducing the source of pathological TDP-43 that could propagate in a prion-like manner.

Alternatively, E3 ubiquitin ligases have also emerged as promising targets for genetic manipulation. Expression of the E3 ubiquitin ligase Znf179 in mouse brains prevented TDP-43 accumulation by stimulating TDP-43 polyubiquitination and enhancing 26S proteasome activity. Conversely, knockout of Znf179 increased the formation of insoluble TDP-43 cytoplasmic inclusions, underscoring the importance of this pathway in TDP-43 homeostasis [173].

Similarly, overexpression of another E3 ubiquitin ligase, Praja-1, has been shown to decrease cytoplasmic TDP-43 CTFs and inclusion formation in cells and mouse motor neurons [174]. Praja-1 appeared to facilitate greater association between pathological TDP-43 and the ubiquitin-conjugating enzyme UBE2E3, thereby increasing TDP-43 ubiquitination and proteasomal targeting [175].

These findings suggest that strategies aimed at increasing the association of pathological TDP-43 with specific UPS components may be more effective than broadly stimulating the abundance of UPS cargo receptors or proteasomal assembly. By facilitating the specific polyubiquitination and proteasomal targeting of pathological TDP-43, these approaches could potentially reduce the pool of misfolded proteins available for further propagation.

It is important to note that while these genetic manipulation strategies show promise in cellular and animal models, translating them into effective therapies for human patients presents significant challenges. Issues such as delivery methods, potential off-target effects, and long-term safety and efficacy need to be thoroughly addressed. Moreover, given the complex nature of TDP-43 pathology, combination therapies that target multiple aspects of TDP-43 proteostasis may ultimately prove most effective in preventing the prion-like spread of pathological TDP-43.

### 9.4. Targeting the Exosome-Mediated Spread of TDP-43

This approach aims to reduce the intercellular transmission of TDP-43 aggregates. Recent research has shed light on the potential of gene therapy approaches targeting exosome biogenesis or release mechanisms as a strategy to mitigate this spread and potentially slow disease progression.

The secretion of exosomes is regulated by various cellular mechanisms, with Rab GTPases playing a critical role in vesicle trafficking and secretion. Certain Rab proteins, such as Rab27A, are known to facilitate exosome release. This understanding has led to the development of gene therapy strategies aimed at downregulating specific Rab GTPases involved in exosome secretion [176,177].

The inhibition of these proteins through gene therapy approaches could therefore aim at decreasing the release of exosomes enriched with pathological TDP-43. The potential benefit of this approach lies in reducing the intercellular transmission of TDP-43 aggregates, thereby slowing the prion-like spread of the disease. However, it is crucial to note that while targeting exosome secretion may seem beneficial for reducing TDP-43 propagation, a balanced approach is necessary.

This caution is made necessary because studies have indicated that exosomal secretion, while facilitating the spread of pathological proteins, also plays a vital role in cellular clearance mechanisms. In fact, inhibition of exosome production has been shown to exacerbate TDP-43 aggregation and disease phenotypes in animal models. This dual role of exosomes in both promoting and clearing pathological proteins highlights the complexity of targeting this mechanism therapeutically [176,177].

Future research in this area should therefore focus on optimizing gene therapy approaches that selectively modulate exosome biogenesis without completely inhibiting it. This could involve fine-tuning the expression levels of Rab GTPases or exploring other pathways involved in exosome formation and release. The goal would be to strike a balance between reducing the spread of pathological TDP-43 and maintaining the beneficial aspects of exosome-mediated protein clearance.

Moreover, combining gene therapy targeting exosome release with other strategies could provide a more comprehensive approach to managing TDP-43 proteinopathies. For instance, pairing these approaches with therapies that enhance autophagy or the ubiquitin–proteasome system could potentially enhance overall cellular clearance mechanisms while mitigating the risks associated with excessive protein aggregation.

Gene therapies targeting exosome biogenesis or release might therefore offer in the future a promising way to reduce prion-like TDP-43 spread in neurodegenerative diseases. However, as already mentioned, the complex role of exosomes in both disease propagation and cellular homeostasis will necessitate careful consideration and further study.

It is important to note that all these approaches are still in the early stages of research, primarily at the preclinical level. The challenge lies in developing delivery methods that can effectively target the relevant cell populations in the central nervous system and achieve sustained therapeutic effects. Moreover, given the complex and multifaceted nature of TDP-43 pathology, combination therapies that address both the initial formation of pathological TDP-43 and its prion-like spread may ultimately prove most effective.

As research progresses, these targeted approaches aimed at disrupting the prion-like spreading of TDP-43 may offer new hope for slowing or halting the progression of devastating neurodegenerative diseases, like ALS and FTD. However, further studies are needed to fully elucidate the mechanisms of TDP-43 spreading and to translate these promising concepts into clinically viable therapies.

## 10. Conclusions

The prion-like propagation of TDP-43 in neurodegenerative disorders has emerged as a critical concept in understanding the progression and pathology of TDP-43 proteinopathies. And Table 5 provides a comprehensive overview of the key findings, implications, and take-home messages regarding the prion-like propagation of TDP-43 in neurodegenerative disorders.

To briefly recapitulate, the evidence for TDP-43 prion-like behavior is substantial and multifaceted. Seed-dependent aggregation of TDP-43 has been demonstrated both in vitro and in vivo, mirroring classical prion behavior. Furthermore, distinct TDP-43 variants exhibit unique biochemical properties affecting their pathogenicity, reminiscent of prion strains. In vivo studies have revealed that TDP-43 pathology spreads through interconnected neural networks in a manner consistent with prion-like propagation. This spread is facilitated by various cellular mechanisms, including axonal transport, extracellular vesicle secretion, and glial cell involvement.

These insights have significant implications for both diagnostic and therapeutic approaches. On the diagnostic front, the TDP-43 seeding amplification assay (TDP43-SAA) shows promise for the early detection and monitoring of TDP-43 pathology in living patients [120]. Similarly, the real-time quaking-induced conversion (RT-QuIC) method offers potential as a diagnostic tool and biomarker for ALS and FTLD [119,178]. These techniques could revolutionize early diagnosis and disease monitoring, potentially enabling earlier interventions.

Therapeutically, several innovative strategies are emerging. Small molecule inhibitors, such as acridine-imidazole derivatives like AIM4, have shown potential in reducing TDP-43 aggregation in preclinical studies [164,165]. Another promising approach involves structural modifications aimed at stabilizing the native TDP-43 conformation, potentially preventing aggregation while preserving normal function. Immunotherapies targeting toxic TDP-43 aggregates and gene therapies modulating TDP-43 expression or function represent exciting possibilities that warrant further exploration [167,168].

Given the widespread involvement of TDP-43 pathology across multiple neurodegenerative disorders, the prospect of TDP-43-targeting therapies as a pan-neurodegenerative approach is compelling. Since TDP-43 pathology is observed in diseases ranging from ALS and FTLD to Alzheimer’s and Huntington’s disease, therapies that mitigate TDP-43 aggregation and propagation could potentially benefit a broader range of neurodegenerative conditions. However, the variable involvement and specific roles of TDP-43 in each disease context mean that such treatments might be more efficacious in certain TDP-43 proteinopathies than others. Thus, while TDP-43-targeted approaches present promising pan-disease potential, further research is required to understand the nuances of TDP-43′s role in diverse neurodegenerative disorders.

Most importantly, the prion-like hypothesis provides a valuable framework for understanding the progressive nature of TDP-43 proteinopathies and offers new targets for therapeutic intervention. It suggests that early detection and intervention may be possible through novel diagnostic techniques based on the prion-like properties of TDP-43. Moreover, targeting the mechanisms of TDP-43 propagation, rather than just its aggregation, may lead to more effective treatment strategies. The observed interplay between TDP-43 and other protein aggregates, such as the prion protein, highlights the complex nature of neurodegenerative diseases and suggests the potential for combination therapies [121].

However, while significant progress has been made, several challenges remain. Further research is needed to elucidate the precise molecular mechanisms governing TDP-43 propagation, including the roles of post-translational modifications and glial cells. The development of more sensitive and specific biomarkers for early diagnosis and disease progression tracking remains a priority. Additionally, designing therapeutic interventions that can disrupt prion-like transmission while maintaining essential TDP-43 functions presents a significant challenge. The translation of promising preclinical strategies into effective clinical interventions will require continued effort and innovation.

In conclusion, the prion-like hypothesis of TDP-43 propagation has reshaped our understanding of TDP-43 proteinopathies and opened new avenues for diagnosis and treatment. As we continue to unravel the complexities of these devastating neurodegenerative disorders, this framework offers hope for developing more effective interventions and, ultimately, improving outcomes for affected individuals. Ongoing research in this field is advancing our understanding and may pave the way for future strategies aimed at not only treating but potentially preventing these conditions. However, while progress is being made, achieving prevention remains a long-term goal, with significant challenges still to be addressed. As our understanding of TDP-43 prion-like behavior advances, we move closer to transformative advancements in the management of these challenging conditions.

## Figures and Tables

**Table 1:** Main post-translational modifications of TDP-43.

Modification	Sites	Function
Phosphorylation	S375, S379, S403, S404, S409, S410	Affects solubility, localization, and protein interactions
Ubiquitination	K48-linked and K63-linked	Involved in protein degradation and aggregation
Acetylation	K84, K136	Affects nuclear localization, RNA binding, and phase separation
SUMOylation	K136	Regulates RNA splicing, cellular distribution, and stress granule association
O-GlcN-Acylation	T199, T233	Regulates TDP-43 function and stability
Cysteine oxidation	C173, C175	Leads to protein aggregation and disease progression
Proteolytic cleavage	Caspase-3, Caspase-7, Caspase-4, AEP	Generates fragments that can contribute to aggregation
Argynilation	C-terminal region	Promotes degradation by the ubiquitin–proteasome system
Citrullination and monomethylation	R293	Not yet functionally characterized

**Table 2:** Evidence for prion-like spreading of TDP-43.

Feature	Evidence
Seed-dependent aggregation	Pre-formed aggregates induce aggregation in cultured cells.
Cell-to-cell transmission	Aggregates spread between cells via microvesicles or exosomes.
Regional progression in the nervous system	Aggregates propagate along neural networks.
Strain-like behavior	Different aggregate conformations influence propagation and pathogenicity.
Tunneling nanotubes and extracellular vesicles	Aggregates are transmitted via TNTs and exosomes.
Interaction with cellular prion protein (PrPC)	PrPC enhances TDP-43 fibril uptake and neurotoxicity.

The references supporting the mechanisms of TDP-43 prion-like propagation are provided in the corresponding subsections of the manuscript, allowing for a detailed exploration of the specific studies and findings associated with each aspect of the prion-like spreading process.

**Table 3:** Mechanisms of TDP-43 prion-like propagation.

Mechanism	Description
Cellular uptake and release	Pathological TDP-43 enters and exits cells through membrane fusion or endocytosis.
Intercellular transport mechanisms	Extracellular vesicle secretion and macropinocytosis facilitate TDP-43 dissemination.
Direct cell-to-cell transmission	Aggregates can move between cells in co-culture.
Role of glial cells	Astrocytes and microglia contribute to TDP-43 spread by uptake and release.
Disruption of axonal transport	TDP-43 aggregates may disrupt axonal transport via CK2 activation.
Sequential progression of pathology	TDP-43 aggregates spread in a manner similar to prions, aligning with disease progression.

The references supporting the mechanisms of TDP-43 prion-like propagation are provided in the corresponding subsections of the manuscript, allowing for a detailed exploration of the specific studies and findings associated with each aspect of the prion-like spreading process.

**Table 4:** Therapeutic strategies for targeting prion-like TDP-43 spreading.

Strategy	Mechanism	Potential Benefits	Challenges
Targeting TDP-43 aggregation	Small molecule inhibitors, structural modifications	Disrupt aggregation, stabilize native conformation, reduce toxic effects	Potential side effects, specificity concerns
Immunotherapy	Antibodies, single-chain variable fragments	Target pathological TDP-43, disrupt harmful protein interactions, reduce neuroinflammation	Delivery to CNS, potential for immune reactions
Intrabody therapy	Intracellularly expressed antibody fragments	Bind and eliminate misfolded TDP-43	Delivery to CNS, potential for off-target effects
Targeting autophagy and UPS components	Genetic manipulation of UPS components, autophagy pathways	Enhance TDP-43 degradation, reduce misfolded TDP-43	Delivery challenges, potential for unintended consequences
Targeting exosome-mediated spread	Gene therapy targeting exosome biogenesis or release	Reduce intercellular transmission of TDP-43	Potential impact on normal cellular functions, balance between reducing spread and maintaining clearance

Strategy: The therapeutic approach employed. Mechanism: The underlying mechanism of action. Potential Benefits: The expected positive outcomes of the strategy. Challenges: Potential drawbacks or limitations to consider.

**Table 5:** Summary of key findings and implications of TDP-43 prion-like propagation in neurodegenerative disorders.

Aspect	Key Points	Take-Home Message
Evidence for prion-like behavior	Seed-dependent aggregation Distinct TDP-43 variants Spread through neural networks Facilitated by transport, vesicles, and glial cells	TDP43 exhibits prion-like characteristics
Diagnostic implications	TDP-43SAA for early detection RTQuIC as a potential biomarker	Prion-like properties enable novel diagnostic techniques
Therapeutic strategies	Small molecule inhibitors Structural modifications Immunotherapies and gene therapies	Targeting propagation mechanisms offers potential for effective treatments
Future challenges	Elucidating molecular mechanisms Developing sensitive biomarkers Designing interventions that maintain TDP-43 function Translating preclinical findings to clinical interventions	Continued research is needed to address challenges and translate findings
Overall impact	Reshaped understanding of TDP-43 proteinopathies Opened new avenues for diagnosis and treatment Potential for transformative advancements	Prion-like hypothesis offers a promising framework for advancing disease management

This table summarizes the main aspects of the TDP-43 prion-like behavior, including evidence supporting the hypothesis, diagnostic and therapeutic implications, future challenges, and overall impact on our understanding of TDP-43 proteinopathies. The take-home messages highlight the most important points to be considered by readers.
